# Granulated Wheat Biscuit

**Published:** 1875-09

**Authors:** 


### GRANULATED WHEAT BISCUIT.

About two months ago some friends of ours organized an association, having for its object the introduction to the public of new and desirable articles of cooked food. They established their office in the same building with us, and announced in our advertising pages the first product of their labors—the Granulated Wheat Biscuit. We tested their granulated food carefully, examined the process of manufacture, and described it in our columns. We spoke of it as good food and predicted for it a large sale.

The prediction is more than verified. The sale may well be deemed enormous. The orders exceed the capacity of the ovens, which now turn out five barrels a day. New ovens are being built, and the baking capacity will be about three tons a week a month hence. If this supply proves inadequate, as appears probable, the company will again enlarge in order to meet the demand. The active demand for this food arises from the superiority of the article and the publicity given thereto by us. The officers of the Health Food Company inform us that they have received orders from all the States and Territories, and some from foreign countries, and that nearly all letters allude to Hall's Journal of Health as the source of the writer's knowledge concerning the food.

There are several peculiarities about this food, which it will be well to remember. The wheat used is the finest and whitest and soundest and plumpest to be found. On the perfection of the grain depend not only the superiority of the food but the success of the various processes which enter its manufacture. The grain is kiln-dried, and is then reduced to a granular powder by pounding in a stamp-mill. This is readily accomplished with good wheat; while imperfect wheat does not bruise or crumble readily, having a tendency to flatten and agglutinate, rather than fall to pieces. No sifting is had, for this would remove a valuable property of the grain. The powder is next mixed by machinery with pure soft water, and after a good deal of kneading and pounding, the dough is passed between steel rollers, and when reduced to a very thin sheet is cut into disks. It is then baked to a delicate shade of brownness in an enormous oven, constructed entirely from soap-stone. The color is very uniform, the heat employed being regulated with thermometrical precision. Not a biscuit is burnt or underdone.

As may be inferred, the bread thus produced is as wholesome, digestible and nutritious as any wheat food can possibly be. It is merely cooked wheat, in the form of a wafer or disk—nothing more. Nothing but water has been added to it, and even that does not remain. Not a trace of salt or grease or dirt can be found by careful analysis.

We have been asked as to its value as food, for persons having feeble digestive powers, and have cheerfully borne testimony to its superiority. To our taste it is an exceedingly palatable bread. Certainly no other cracker can compare with it in this respect, nor does any cracker which we have seen at all approach it in healthfulness and strength-giving power.

Invalids, and persons recovering from sickness, seem to take to this food wonderfully. It is hard and dry, but it is thin and crispy as well, and therefore it is easily reduced to pulp in the mouth. It cannot be swallowed without complete mastication, and this fact makes it a valuable food for children. If eaten without being soaked or softened, save by the saliva, it is certain to be well fitted for the stomach when it enters there.

Of course hard food is better than soft. It is better for the teeth and gums, because the exercise and attrition attending its mastication, assist in strengthening the dental organs. The jaws must have the gymnastics of chewing solid food, or their power and strength is lessened, and they grow narrow and feeble. If our young folks could live on these hard, dry, crisp biscuits for a few years of life, we should see finer formed faces, more perfect jaws, few if any crowded and irregular teeth, and rarely if ever a decayed or disfigured one. For not only would this excellent food supply plump flesh, strong muscle and sound bone, but its very hardness would give that salutary exercise to the organs involved in mastication, which is so essential to beauty of color and outline. For round and rosy cheeks, depend more upon suitable food than upon any other single condition.

On the whole, we look upon these Granulated Wheat Biscuits as the best food at present offered for sale. With less than a pound of these daily, and a little sweet milk or butter or cheese, any life, however laborious, may be sustained in full vigor, and in buoyant health. With such a strong and simple diet, good digestion will surely wait on appetite and health on both. For infants and growing children, it is probably the best food in the world to-day.

